# Randomized phase II study of the PDGFRα antibody olaratumab plus liposomal doxorubicin versus liposomal doxorubicin alone in patients with platinum-refractory or platinum-resistant advanced ovarian cancer

**DOI:** 10.1186/s12885-018-5198-4

**Published:** 2018-12-27

**Authors:** William P. McGuire, Richard T. Penson, Martin Gore, Antonio Casado Herraez, Patrick Peterson, Ashwin Shahir, Robert Ilaria

**Affiliations:** 1 Virginia Commonwealth University, 1201 E Marshall St, Room 11-210, Richmond, VA 23298 USA; 2 Massachusetts General Hospital, Yawkey 9-064, 32 Fruit St, Boston, MA 02114 USA; 3 The Royal Marsden Hospital, Fulham Road, London, SW3 6JJ UK; 4 Hospital Clinico, San Carlos Servicio de Oncologia Medica, Madrid, Spain; 5 Eli Lilly and Company, Indianapolis, IN USA; 6 Eli Lilly and Company, Lilly UK, EMC Building, Erl Wood Manor, Windlesham, Surrey, GU20 6PH UK; 7 Celgene Corporation, 86 Morris Ave, Summit, NJ 07901 USA

**Keywords:** Ovarian cancer, Olaratumab, Liposomal doxorubicin, Platinum refractory, Platinum resistant

## Abstract

**Background:**

Olaratumab is a platelet-derived growth factor receptor-α (PDGFRα)-targeting monoclonal antibody blocking PDGFRα signaling. PDGFRα expression is associated with a more aggressive phenotype and poor ovarian cancer outcomes. This randomized, open label phase II study evaluated olaratumab plus liposomal doxorubicin compared with liposomal doxorubicin alone in advanced ovarian cancer patients.

**Methods:**

Patients with platinum-refractory or platinum-resistant advanced ovarian cancer were randomized 1:1 to receive liposomal doxorubicin (40 mg/m^2^, intravenous infusion) administered every 4 weeks with or without olaratumab (20 mg/kg, IV infusion) every 2 weeks. Patients were stratified based on prior response to platinum therapy (refractory vs resistant). The primary efficacy endpoint was progression-free survival (PFS). Secondary endpoints included overall survival (OS), objective response rate, duration of response, and safety.

**Results:**

A total of 123 patients were treated (62 olaratumab+liposomal doxorubicin; 61 liposomal doxorubicin). Median PFS was 4.2 months for olaratumab+liposomal doxorubicin and 4.0 months for liposomal doxorubicin (stratified hazard ratio [HR] = 1.043; 95% confidence interval [CI] 0.698–1.558; *p* = 0.837). Median OS was 16.6 months and 16.2 months in the olaratumab+liposomal doxorubicin and liposomal doxorubicin arms, respectively (HR = 1.098; 95% CI 0.71–1.71). In the platinum-refractory subgroup, median PFS was 5.5 months (95% CI 1.6–9.2) and 3.7 months (95% CI 1.9–9.2) in the olaratumab+liposomal doxorubicin (*n* = 15) and liposomal doxorubicin arms (*n* = 16), respectively (HR = 0.85; 95% CI 0.38–1.91). Overall, 59.7% (olaratumab+liposomal doxorubicin) and 65.6% (liposomal doxorubicin) of patients reported grade ≥ 3 adverse events regardless of causality. The most common treatment-emergent adverse events (all grades) regardless of causality were fatigue related (61%), nausea (57%), and constipation (52%) with olaratumab+liposomal doxorubicin and nausea (64%), fatigue related (62%), and mucositis (46%) with liposomal doxorubicin.

**Conclusions:**

The addition of olaratumab to liposomal doxorubicin did not result in significant prolongation of PFS or OS in platinum-resistant or platinum-refractory ovarian cancer.

**Trial registration:**

ClinicalTrials.gov identifier: NCT00913835; registered June 2, 2009.

## Background

Ovarian cancer is a family of many diseases, each with specific histology, risk factors, molecular characteristics, and treatment [1]. Epithelial ovarian cancer (EOC) comprises 90% of cases; of these, serous is the most common subtype [1]. The current standard treatment for EOC of all subtypes involves debulking surgery followed by combination chemotherapy with a platinum plus taxane base [2–4]. Patients who relapse after first-line treatment may be classified into 1 of 2 subgroups: those with platinum-refractory/−resistant disease and those with platinum-sensitive disease [5]. Although many agents are available for patients with platinum-resistant or -refractory disease who have also received first-line paclitaxel, there is still no definitive second-line treatment for these patients [2–4].

Several phase II clinical studies of patients with platinum-resistant or -refractory disease have demonstrated the benefit of using doxorubicin in combination therapy with other agents [6–12]. Liposomal doxorubicin is approved by the United States Food and Drug Administration and the European Medicine Agency for ovarian cancer in women who have failed platinum-based chemotherapy [13, 14]. Treatment guidelines recognize that combining traditional chemotherapeutic agents with drugs targeting growth factors/receptors may be more effective for treating platinum-resistant/−refractory recurrent ovarian cancer than chemotherapy alone [3, 4].

The platelet-derived growth factor receptors (PDGFRs: PDGFRα and PDGFRβ) are transmembrane receptor tyrosine kinases that are activated by their cognate ligands [15]. Platelet-derived growth factor (PDGF)-AA binds PDGFRα, whereas PDGF-AB and PDGF-BB recognize both PDGFRα and PDGFRβ [15]. Upon binding of circulating PDGF ligand, PDGFRα and β subunits homodimerize or heterodimerize, undergo autophosphorylation, and activate downstream signal transduction molecules, including phosphoinositide 3-kinase, Ras, phospholipase Cγ, and Src [16, 17]. PDGFR signaling plays a significant part in mesenchymal biology, including mesenchymal stem cell differentiation, proliferation, and angiogenesis [18, 19]. Aberrant PDGF/PDGFR signaling is involved in the development and maintenance of cancer, and has been implicated in modulating the tumor or stromal microenvironment thus facilitating metastasis in several malignancies [16, 17]. The PDGF/PDGFR axis has pro-angiogenic activity and may contribute to resistance to anti-vascular endothelial factor therapy [20].

Expression of PDGFRα has been reported in ovarian cancers, although the prevalence varies [21–23]. This may reflect the variety of methods and reagents used to measure PDGFRα, with recent reports suggesting that some of the reagents used in previous studies may be nonspecific for PDGFRα [24]. A study by Matsuo et al. [25] on the extent of PDGFRα protein expression assessed in 176 human ovarian tumors revealed that PDGFRα expression was significantly associated with serous histology (serous vs nonserous, 77% vs 46%, respectively; odds ratio, 4.0) and advanced stage (odds ratio, 1.7). The most common type of histology was high-grade serous ovarian carcinomas [25]. Among patients with high-grade serous tumors, PDGFRα-expressing tumors were associated with significantly poorer survival outcomes (median OS, 51 months) compared to patients with PDGFRα-nonexpressing tumors (median OS, 174 months; *p* = 0.014) [25]. In addition, when controlled for age and stage, PDGFRα expression remained a significant variable for OS [25].

When present, PDGFRα may be stimulated in an autocrine loop by ovarian tumors co-expressing PDGF-AB [23]. This activation induced Akt- and mitogen-activated protein kinase (MAPK) -mediated proliferation of tumor cells [23]. In a clinical trial of patients who were platinum-resistant or -refractory, the PDGFR kinase inhibitor, imatinib, in combination with docetaxel showed an objective response rate (ORR) of 22% (5 of 23 patients) [26].

Olaratumab (LY3012207; formerly IMC-3G3) is a recombinant fully human immunoglobulin G subclass 1 (IgG1) monoclonal antibody that specifically binds to PDGFRα, blocking signaling of PDGF ligands [27]. The antibody inhibits PDGFR ligand–induced receptor autophosphorylation and phosphorylation of downstream signal transduction via Akt and MAPK [27]. Olaratumab has antitumor activity in in vivo tumor models thought to be driven by a PDGF-PDGFRα autocrine loop [27]. In mouse models of pediatric osteosarcoma and malignant rhabdoid tumor, olaratumab delayed tumor growth, and this activity was enhanced by chemotherapy (cisplatin or doxorubicin) [28]. Likewise, olaratumab alone and in combination with docetaxel significantly reduced tumor weight in in vivo xenograft models of ovarian carcinoma compared to control and docetaxel alone, respectively [25]. In a phase Ib/IIa study, the combination of olaratumab+doxorubicin significantly improved both progression-free survival (PFS; 6.6 vs 4.1 months in phase II) and overall survival (OS; 26.5 vs 14.7 months in phase II) relative to doxorubicin alone in patients with advanced soft tissue sarcoma [29]. The present phase II study was performed to evaluate the combination of olaratumab+liposomal doxorubicin vs liposomal doxorubicin alone in patients with platinum-refractory or platinum-resistant advanced ovarian cancer.

## Methods

### Study design and patient enrollment

This was a randomized, open-label, multicenter phase II study. The primary endpoint was PFS. Secondary endpoints included ORR, OS, duration of response, and safety. This study (NCT00913835) was conducted according to the Declaration of Helsinki and with approval from Institutional Review Boards of all participating study sites. All participants provided written informed consent prior to any study-related procedures. This manuscript adheres to CONSORT reporting guidelines.

### Study participants

This study enrolled adult females (≥18 years) with histologically or cytologically confirmed EOC, primary peritoneal carcinoma or fallopian tube cancer that was platinum-resistant or platinum-refractory. Patients must have completed 1 to 3 platinum-containing regimens for their disease. Platinum-refractory was defined as progression or persistent disease while receiving platinum-containing therapy; platinum-resistant was defined as disease recurrence ≤12 months following platinum-containing chemotherapy.

Additional enrollment criteria included measurable disease according to the Response Evaluation Criteria in Solid Tumors (RECIST v1.0) guidelines and an Eastern Cooperative Oncology Group performance status (ECOG PS) score of ≤1. Prior to enrollment, patients had to recover to grade ≤ 1 from the effects of prior therapies according to National Cancer Institute Common Terminology Criteria for Adverse Events, Version 3.0 (NCI-CTCAE v3.0), with the exception of peripheral neuropathy (which must resolve to grade ≤ 2). Patients with brain metastases, leptomeningeal disease, or increased level of cancer antigen 125 (CA125) in the absence of concomitant clinical or radiographic progression were excluded.

### Study procedures

**Fig. 1:**
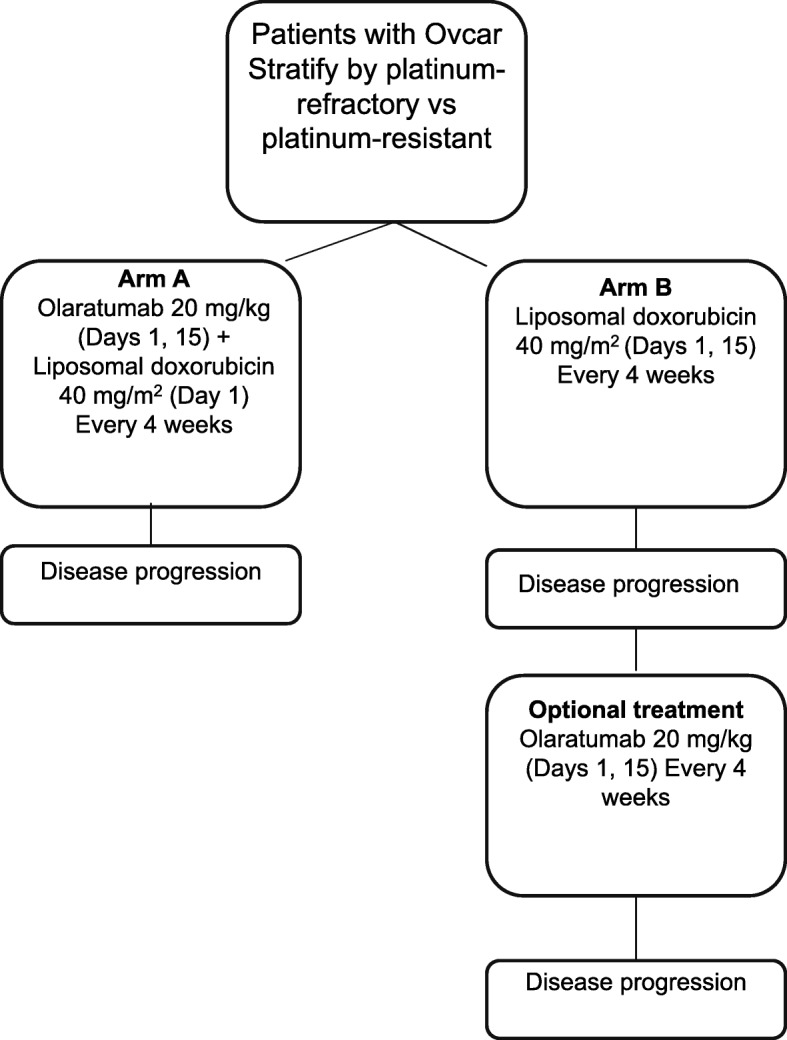
Study design: open-label, nonblinded, multicenter, Phase II trial

Study site personnel randomized patients using either a call-in Interactive Voice Response System (IVRS) or Interactive Web Response System (IWRS). The IVRS/IWRS assigned a unique identification number to each patient. Patients were stratified based on the previous response to platinum therapy (refractory vs resistant). Within each stratum, patients were randomly assigned (1:1) to olaratumab+liposomal doxorubicin or liposomal doxorubicin alone. Patients in the olaratumab+liposomal doxorubicin arm received IV olaratumab at 20 mg/kg on days 1 and 15 and liposomal doxorubicin at 40 mg/m^2^ on day 1 of a 28-day cycle; patients in the liposomal doxorubicin arm received 40 mg/m^2^ on day 1 every 4 weeks (Fig. 1). Upon disease progression, patients in the liposomal doxorubicin arm could elect to receive olaratumab monotherapy (20 mg/kg every 2 weeks); data from this group are not presented due to small sample size.

Patients underwent radiographic disease assessment approximately every 8 weeks. Treatment was continued until disease progression, unacceptable toxicity, protocol noncompliance, or consent withdrawal. After the last study visit, follow-up information on additional anticancer treatment, disease progression, and survival was collected every 2 months for up to 2 years.

A safety review by the Safety Review Committee (SRC) was mandated after 6 patients were treated for at least 8 weeks on the liposomal doxorubicin+olaratumab arm. Any patient who discontinued for toxicity prior to 8 weeks was also included in the review. If the incidence of serious adverse events (SAE) markedly exceeded that expected of liposomal doxorubicin monotherapy, protocol modification, termination, or ongoing monitoring was considered by the SRC. There were no interim efficacy analysis performed for this study.

### Statistical analyses

A total of 110 evaluable patients were planned with 55 patients for each treatment arm. This sample size would provide a 69.3% power to detect an expected increase of median PFS from 12 weeks (liposomal doxorubicin monotherapy) to 18.5 weeks when liposomal doxorubicin was combined with olaratumab. Final analysis was planned to be performed when at least 99 PFS events were observed or all patients discontinued from study therapy. Additional assumptions included an accrual time of 55 weeks at a rate of 2 patients per week, a follow-up time of 28 weeks, and an α-level of 10% using a 2-sided test.

Efficacy data for the primary endpoint, which was PFS, and the secondary endpoints were analyzed in the modified intent-to-treat (mITT) population, which included all patients who were randomized and received any quantity of study drug; patients were categorized by the treatment arm to which they were randomized, regardless of the actual treatment received. For the primary endpoint, PFS, the Kaplan-Meier method was used to estimate the median PFS time, together with a 95% confidence interval (CI). Comparison between arms was performed using the stratified log rank test, and hazard ratios (HRs) were determined by a Cox proportional hazards regression model. The log rank test and Cox regression models were used to compare the survival curves adjusting for prior response to platinum treatment as a stratification factor (platinum-refractory vs platinum-resistant). Sensitivity analyses for PFS using various censoring rules were defined in the separate statistical analysis plan. Additional analysis of PFS was performed for treatment effects across different subgroups. The subgroups were defined by the stratification factor (platinum-refractory, platinum-resistant), age (≤62 years, > 62 years), ECOG PS (0, > 0), progressive disease (PD) in previous anticancer therapy (yes, no), duration of disease (≤15.21 months, > 15.21 months), and serum levels of CA125 (≤64.3 kU/L, > 64.3 kU/L).

Safety analysis was conducted on all patients who received any quantity of olaratumab, regardless of study eligibility (safety population). Patients were categorized by the treatment actually received, regardless of the arm to which they were randomized. An adverse event (AE) was regarded as treatment-emergent if its onset date occurred any time after the administration of the first dose of study drug and up to 30 days after the last dose of study treatment (or up to any time if related to study treatment), or if it occurred prior to first dose date and worsened while on therapy.

## Results

### Demographics and disposition

This study was conducted at 22 study sites in 3 countries (United States, United Kingdom, and Spain) between 11 June 2009 (first patient visit) and 13 February 2014 (last patient visit). The mITT population comprised 123 patients. Of these, the majority were White (87.8%) (Table 1). The median age was 59.0 years (range, 34–83 years). The patients had an ECOG PS of either 0 (55.3%) or 1 (44.7%) at study entry. Overall, 75.6% of patients were platinum-resistant, whereas 24.4% were platinum-refractory. Following disease progression, 28 patients in the liposomal doxorubicin arm elected to receive olaratumab monotherapy.

**Table 1:** Baseline demographics

	Olaratumab + Liposomal Doxorubicin (*n* = 62)	Liposomal Doxorubicin (*n* = 61)	Total (*N* = 123)
Sex, No. (%)			
Female	62 (100.0)	61 (100.0)	123 (100.0)
Race, No. (%)			
Asian	1 (1.6)	3 (4.9)	4 (3.3)
Black or African American	5 (8.1)	1 (1.6)	6 (4.9)
Native Hawaiian or other Pacific Islander	0	1 (1.6)	1 (0.8)
White	54 (87.1)	54 (88.5)	108 (87.8)
Other	2 (3.2)	2 (3.3)	4 (3.3)
Ethnicity, No. (%)			
Hispanic or Latino	1 (1.6)	6 (9.8)	7 (5.7)
Non-Hispanic or Latino	61 (98.4)	54 (88.5)	115 (93.5)
Missing	0	1 (1.6)	1 (0.8)
Age, yrs			
Mean (SD)	58.7 (10.07)	59.8 (9.70)	59.3 (9.86)
ECOG PS, No. (%)			
0	37 (59.7)	31 (50.8)	68 (55.3)
1	25 (40.3)	30 (49.2)	55 (44.7)
Prior chemotherapy, No. (%)	62 (100.0)	61 (100.0)	123 (100.0)
Stratification factor (CRF), No. (%)			
Platinum-refractory	13 (21.0)	17 (27.9)	30 (24.4)
Platinum-resistant	49 (79.0)	44 (72.1)	93 (75.6)
Stratification factor (IVRS), No. (%)			
Platinum-refractory	15 (24.2)	16 (26.2)	31 (25.2)
Platinum-resistant	47 (75.8)	45 (73.8)	92 (74.8)

*CRF* case report form, *ECOG PS* Eastern Cooperative Oncology Group performance status, *IVRS* interactive voice response system, *SD* standard deviation, *yrs*. years

Of 135 patients who entered the study, 125 were randomized and 123 were treated (62 olaratumab+liposomal doxorubicin, 61 liposomal doxorubicin) (Table 2). Two patients were randomized but not treated: One patient assigned to the olaratumab+liposomal doxorubicin arm discontinued for an unknown reason, and one patient assigned to the liposomal doxorubicin arm was not treated due to withdrawal by the patient. A total of 121 patients (61 in the olaratumab+liposomal doxorubicin arm, 60 in the liposomal doxorubicin arm) completed the study (Table 2). At the time of database lock, 2 patients were still on study therapy or on study evaluations. Fifty-four patients (43.9%) discontinued study therapy because of progressive disease per RECIST, 18 patients (14.6%) discontinued therapy because of symptomatic deterioration, and 2 patients (1.6%) in the olaratumab+liposomal doxorubicin arm died. Both deaths occurred ≥21 days after last dose of study treatment (27 and 21 days after the last olaratumab dose). One patient died due to progressive disease and the other due to pulmonary embolism considered by the investigator to be possibly related to study treatment. Nine patients (7.3%) discontinued the study therapy due to AEs.

**Table 2:** Patient disposition

	No. (%) of Patients		
	Olaratumab + Liposomal Doxorubicin	Liposomal Doxorubicin	Total
mITT population	62	61	123
Treated	62 (100.0)	61 (100.0)	123 (100.0)
On treatment^a^	1 (1.6)	0	1 (0.8)
Off treatment	61 (98.4)	61 (100.0)	122 (99.2)
Reasons for discontinuation of study therapy			
Adverse event	2 (3.2)	7 (11.5)	9 (7.3)
Death	2 (3.2)	0	2 (1.6)
PD per RECIST	42 (67.7)	12 (19.7)	54 (43.9)
PD, symptomatic deterioration	10 (16.1)	8 (13.1)	18 (14.6)
Withdrawal by patient	1 (1.6)	3 (4.9)	4 (3.3)
Lost to follow-up	1 (1.6)	0	1 (0.8)
Other	3 (4.8)	3 (4.9)	6 (4.9)
Reasons for discontinuation for patients electing to receive olaratumab monotherapy after progression on liposomal doxorubicin			
PD per RECIST	0	26 (42.6)	26 (21.1)
PD, symptomatic deterioration	0	2 (3.3)	2 (1.6)
On study^a^	1 (1.6)	1 (1.6)	2 (1.6)
Off study	61 (98.4)	60 (98.4)	121 (98.4)

mITT, modified intent-to-treat; PD, progressive disease; RECIST, Response Evaluation Criteria in Solid Tumors.^a^Refers to those who were still on study therapy or on study evaluations as of cutoff date. For patient who discontinued study therapy for reasons other than PD, radiological scans continued until a documented PD. After PD was documented, patient was considered off study. Patients were followed for survival status. In any study phase, patients could withdraw consent or become lost to follow-up

### Efficacy

**Fig. 2:**
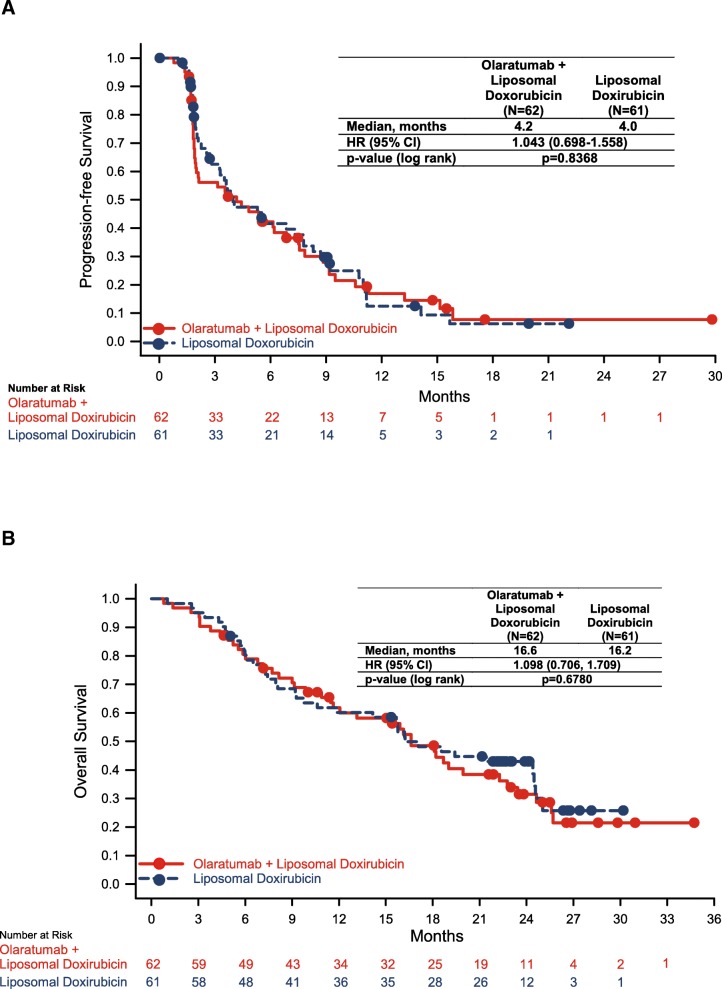
Kaplan-Meier plots of progression-free (**a**) and overall (**b**) survival

Forty-nine patients (79.0%) in the olaratumab+liposomal doxorubicin arm and 47 patients (77.0%) in the liposomal doxorubicin arm had a total of 96 PFS events. Median PFS was similar between groups (stratified HR = 1.043; *p* = 0.837) (Fig. 2a). The 1-year PFS rate was 16.9% in the olaratumab+liposomal doxorubicin arm and 12.5% in the liposomal doxorubicin arm.

In the platinum-refractory subgroup, median PFS appeared slightly longer in the olaratumab+liposomal doxorubicin arm than in the liposomal doxorubicin arm (5.5 months vs 3.7 months [HR = 0.85; 95% CI 0.38–1.91]) (Table 3). In the platinum-resistant subgroup, median PFS was similar between groups (3.7 months in the olaratumab+liposomal doxorubicin arm vs 4.0 months in the liposomal doxorubicin arm; [HR = 1.13; 95% CI 0.71–1.80]) (Table 3).

**Table 3:** Subgroup analysis of progression-free survival

	Olaratumab + Liposomal Doxorubicin (*n* = 62)				Liposomal Doxorubicin(*n* = 61)				Hazard Ratio^a^	
	No.	Events	Median, months^b^	95% CI^b^	No.	Events	Median, months^b^	95% CI^b^	HR	95% CI
Stratification factor (from IVRS)										
Platinum-refractory	15	12	5.5	(1.6–9.2)	16	13	3.7	(1.9–9.2)	0.85	(0.38–1.91)
Platinum-resistant	47	37	3.7	(1.9–6.2)	45	34	4.0	(2.7–7.8)	1.13	(0.71–1.80)

CI, confidence interval; IVRS, interactive voice response system

^a^Hazard ratio is expressed as olaratumab+liposomal doxorubicin/liposomal doxorubicin and estimated from Cox model

^b^Estimated by the Kaplan-Meier method

Subgroup analysis showed that patients with disease duration of less than 15.2 months had improvement in PFS with olaratumab+liposomal doxorubicin treatment (HR = 0.57; 95% CI 0.29–1.12) (*n* = 50) compared with patients in the liposomal doxorubicin arm. Likewise, patients with a lower CA125 (≤64.3) had higher PFS with olaratumab+liposomal doxorubicin treatment compared with patients in the liposomal doxorubicin arm, achieving an HR of 0.5 (95% CI 0.21–1.22) (*n* = 27). It should be noted that the 95% CIs for all considered subgroups covered a HR of 1.0, indicating no significant treatment difference on PFS between the 2 treatment arms.

For the secondary endpoints, a total of 44 OS events (censored) were observed across both study arms, including 21 (33.9%) in the olaratumab+liposomal doxorubicin arm and 23 (37.7%) in the liposomal doxorubicin arm. Median OS was similar between groups (HR = 1.098; 95% CI 0.71–1.71; *p* = 0.678) (Fig. 2b). The 1-year survival was 61.8% for patients receiving olaratumab+liposomal doxorubicin and 60.2% for patients treated with liposomal doxorubicin. The 2-year survival was 31.5 and 42.9% in the olaratumab+liposomal doxorubicin and liposomal doxorubicin arms, respectively. No statistically significant difference in OS was observed between the treatment groups.

With respect to overall tumor response, there were no complete responses (CRs) in either arm. The ORR (CR + partial response [PR]) was 12.9 and 16.4% in the olaratumab+liposomal doxorubicin and liposomal doxorubicin arms, respectively. The disease control rate (CR + PR + stable disease) was 56.5% in the olaratumab+liposomal doxorubicin arm and 63.9% in the liposomal doxorubicin arm. Median duration of response was 39.1 weeks and 16.9 weeks in the olaratumab+liposomal doxorubicin and liposomal doxorubicin arms, respectively.

### Safety

Patients received a median of 4 cycles for each regimen (range 1, 24 for olaratumab+liposomal doxorubicin arm; range 1, 15 for liposomal doxorubicin arm). Of the 123 treated patients in this study, 41 (66%) in the olaratumab+liposomal doxorubicin arm and 38 (62%) in the liposomal doxorubicin arm had died at the time of data cutoff, disease progression being the most common cause of death (35 olaratumab+liposomal doxorubicin, 33 liposomal doxorubicin)**.** Three patients (4.8%) in the olaratumab+liposomal doxorubicin arm (1 SAE of disease progression, 1 SAE of intracranial hemorrhage, and 1 SAE of pulmonary embolism) and 2 patients (3.3%) in the liposomal doxorubicin arm (2 SAEs of disease progression) died due to an AE. Three deaths in each treatment arm were from other causes.

The rate of discontinuation due to AEs was higher in the liposomal doxorubicin arm (3.2% vs 11.5%). The only reasons for discontinuation observed in more than 1 patient were pulmonary embolism and mucositis, both in patients in the liposomal doxorubicin arm. The incidence of AEs (all grades and grade ≥ 3) was similar between the olaratumab+liposomal doxorubicin arm and the liposomal doxorubicin arm (all grades, 100% vs 100%; grade ≥ 3, 60% vs 66%; any SAE, all grades, 43.5% vs 37.7%). The most common treatment-emergent adverse events (TEAEs), regardless of causality (with a ≥ 5% between-arm difference), were fatigue- related (61%), nausea (57%), and constipation (52%) with olaratumab+liposomal doxorubicin and nausea (64%), fatigue-related (62%), and mucositis (46%) with liposomal doxorubicin (Table 4).

**Table 4:** Treatment-emergent adverse events, regardless of causality

	No. (%)			
	Olaratumab + Liposomal Doxorubicin (*n* = 62)		Liposomal Doxorubicin (*n* = 61)	
	All grades	Grade ≥ 3	All grades	Grade ≥ 3
Patients with any TEAE	62 (100.0)	37 (59.7)	61 (100.0)	40 (65.6)
Consolidated TEAE category^a^				
Fatigue^b^	38 (61.3)	7 (11.3)	38 (62.3)	1 (1.6)
Mucositis^c^	30 (48.4)	0	28 (45.9)	4 (6.6)
Rash^d^	27 (43.5)	3 (4.8)	18 (29.5)	5 (8.2)
Abdominal pain^e^	24 (38.7)	2 (3.2)	30 (49.2)	8 (13.1)
Neutropenia^f^	20 (32.3)	8 (12.9)	13 (21.3)	5 (8.2)
Neuropathy^g^	12 (19.4)	0	9 (14.8)	0
Hypomagnesemia^h^	10 (16.1)	0	6 (9.8)	1 (1.6)
Preferred terms^a,i^				
Nausea	35 (56.5)	2 (3.2)	39 (63.9)	1 (1.6)
Constipation	32 (51.6)	2 (3.2)	24 (39.3)	0
Vomiting	21 (33.9)	3 (4.8)	20 (32.8)	6 (9.8)
Palmar-plantar erythrodysesthesia syndrome	21 (33.9)	7 (11.3)	27 (44.3)	4 (6.6)
Diarrhea	19 (30.6)	2 (3.2)	13 (21.3)	0
Back pain	16 (25.8)	1 (1.6)	10 (16.4)	1 (1.6)
Abdominal distension	15 (24.2)	2 (3.2)	6 (9.8)	2 (3.3)
Urinary tract infection	15 (24.2)	0	5 (8.2)	2 (3.3)
Headache	12 (19.4)	0	7 (11.5)	1 (1.6)
Anemia	10 (16.1)	3 (4.8)	13 (21.3)	1 (1.6)
Dysgeusia	10 (16.1)	0	3 (4.9)	0
Dehydration	9 (14.5)	3 (4.8)	3 (4.9)	2 (3.3)
Weight decreased	8 (12.8)	0	4 (6.6)	0
Proteinuria	7 (11.3)	0	2 (3.3)	0
Muscle spasms	7 (11.3)	0	3 (4.9)	0
Pain in extremity	4 (6.5)	0	9 (14.8)	1 (1.6)
TEAE of special interest				
Infusion-related reactions^j^	6 (9.7)	0	3 (4.9)	0
Any SAE	27 (43.5)	21 (33.9)	23 (37.7)	20 (32.8)
Discontinuation due to TEAE	2 (3.2)	n.r.	7 (11.5)	n.r.

AE, adverse event; MedDRA, Medical Dictionary for Regulatory Activities; n.r., not reported; SAE, serious adverse event; TEAE, treatment-emergent adverse event

^a^TEAEs occurring in ≥10% of patients (all grades) and with a ≥ 5% between-arm difference (all grades or grade ≥ 3)

^b^Consolidated term comprising the following synonymous MedDRA preferred terms: fatigue and asthenia

^c^Consolidated term comprising the following synonymous MedDRA preferred terms: aphthous stomatitis, mucosal inflammation, oropharyngeal pain, and stomatitis

^d^Consolidated term comprising the following synonymous MedDRA preferred terms: rash, rash follicular, rash generalized, rash macular, rash papular, rash pruritic, and rash pustular

^e^Consolidated term comprising the following synonymous MedDRA preferred terms: abdominal pain, abdominal pain lower, and abdominal pain upper

^f^Consolidated term comprising the following synonymous MedDRA preferred terms: leukopenia, neutropenia, neutrophil count decreased, and white blood cell count decreased

^g^Consolidated term comprising the following synonymous MedDRA preferred terms: hypoesthesia, neuropathy peripheral, paraesthesia, and peripheral sensory neuropathy

^h^Consolidated term comprising the following synonymous MedDRA preferred terms: blood magnesium decreased, hypomagnesemia, and magnesium deficiency

^i^Omits preferred terms that are included in consolidated categories

^j^Infusion-related reactions include a combination of specific preferred terms such as infusion-related reactions, anaphylaxis, and signs and symptoms such as flushing and itching

There were no cases of febrile neutropenia in either treatment arm. The rate of serious olaratumab-related infections in the olaratumab+liposomal doxorubicin arm was 1.6%, as was the rate of liposomal doxorubicin–related infections in the liposomal doxorubicin arm.

## Discussion

This study did not meet the primary endpoint of achieving a statistically significant improvement of PFS in the olaratumab+liposomal doxorubicin arm compared with liposomal doxorubicin alone in patients with advanced ovarian cancer. No statistically significant improvement in OS was achieved either, as a secondary endpoint of this study.

In general, safety profiles were similar between treatment arms, and AEs for olaratumab+liposomal doxorubicin were manageable and could be monitored easily. The higher incidence of neutropenia in the olaratumab+liposomal doxorubicin arm did not result in more febrile neutropenia.

There are limitations to this study. Although pretreatment tissue was collected in this study, the diagnostic antibody used to detect PDGFRα expression was subsequently found to have poor specificity for PDGFRα by also detecting PDGFRß [24], precluding any meaningful analysis of PDGFRα status of tissue samples. There were more discontinuations due to progressive disease according to RECIST criteria in the olaratumab+liposomal doxorubicin arm than in the liposomal doxorubicin arm. In addition to the possibility that this was a chance finding, this could reflect bias favoring patients in the investigational arm staying in this unblinded study until formal RECIST criteria for progression were met. This might also explain why discontinuation for toxicity occurred more frequently in the liposomal doxorubicin arm. Nonetheless, most of the AEs observed in this study are consistent with the known safety profile of liposomal doxorubicin or occur in the metastatic ovarian cancer population.

## Conclusions

There was no statistically significant difference between the olaratumab+liposomal doxorubicin arm and the liposomal doxorubicin arm in PFS or secondary endpoints of OS and ORR. Olaratumab given in combination with liposomal doxorubicin was well tolerated.

## Abbreviations

AE: Adverse event
CA125: Cancer antigen 125
CI: Confidence interval
CR: Complete response
ECOG PS: Eastern Cooperative Oncology Group performance status
EOC: Epithelial ovarian cancer
HR: Hazard ratio
IgG1: Immunoglobulin G subclass 1
IV: Intravenous
IVRS: Interactive Voice Response System
IWRS: Interactive Web Response System
MAPK: Mitogen-activated protein kinase
mITT: Modified intent-to-treat
NCI-CTCAE: National Cancer Institute Common Terminology Criteria for Adverse Events
ORR: Objective response rate
OS: Overall survival
PD: Progressive disease
PDGF: Platelet-derived growth factor
PDGFR: Platelet-derived growth factor receptor
PFS: Progression-free survival
PR: Partial response
RECIST: Response Evaluation Criteria in Solid Tumors
SAE: Serious adverse event
SRC: Safety Review Committee
